# Supplementary material for: Lung Auscultation Using the Smartphone—Feasibility Study in Real-World Clinical Practice
Source: Sensors (Basel). 2021 Jul 20;21(14):4931. doi: 10.3390/s21144931 (PMC8309818; doi:10.3390/s21144931)
Supplement: Supplementary file 1 [file sensors-21-04931-s001.zip › sensors-1257421-supplementary.pdf]

sensors

MDPI

**Table S1.** Inter-rater agreement regarding the quality classification and the presence of adventitious sounds by location, age group and diagnostic group.

| | | | Agreement % | Cohens' k | 95% CI | p |
|---|---|---|---|---|---|---|
| Quality | Location | Trachea (n=272) | 97 | 0.88 | 0.81 – 0.95 | <0.001 |
| | | Right Anterior Chest (n=262) | 93 | 0.84 | 0.77 – 0.91 | <0.001 |
| | | Right Posterior Base (n=267) | 91 | 0.81 | 0.74 – 0.88 | <0.001 |
| | | Left Posterior Base (n=259) | 90 | 0.77 | 0.69 – 0.85 | <0.001 |
| | Age group | Children (n=710) | 93 | 0.80 | 0.75 – 0.85 | <0.001 |
| | | Adults (n=350) | 93 | 0.86 | 0.80 – 0.92 | <0.001 |
| | Diagnostic group | Cystic Fibrosis (n=354) | 92 | 0.80 | 0.73 – 0.87 | <0.001 |
| | | Other Respiratory Diseases (n=309) | 81 | 0.93 | 0.88 – 0.98 | <0.001 |
| | | Asthma (n=272) | 88 | 0.73 | 0.64 – 0.82 | <0.001 |
| | | No Respiratory Diseases (n=125) | 94 | 0.85 | 0.74 – 0.96 | <0.001 |
| | | Total (n=1060) | 93 | 0.82 | 0.78 – 0.86 | <0.001 |
| Presence of adventitious sounds | Location | Trachea (n=223) | 93 | 0.72 | 0.59 – 0.85 | <0.001 |
| | | Right Anterior Chest (n=189) | 87 | 0.54 | 0.38 – 0.70 | <0.001 |
| | | Right Posterior Base (n=173) | 91 | 0.67 | 0.52 – 0.82 | <0.001 |
| | | Left Posterior Base (n=184) | 93 | 0.72 | 0.57 – 0.87 | <0.001 |
| | Age group | Children (n=531) | 90 | 0.61 | 0.52 – 0.70 | <0.001 |
| | | Adults (n=238) | 93 | 0.75 | 0.64 – 0.86 | <0.001 |
| | Diagnostic group | Cystic Fibrosis (n=273) | 90 | 0.41 | 0.22 – 0.60 | <0.001 |
| | | Other Respiratory Diseases (n=231) | 90 | 0.87 | 0.80 – 0.94 | <0.001 |
| | | Asthma (n=183) | 87 | 0.38 | 0.18 – 0.58 | <0.001 |
| | | No Respiratory Diseases (n=92) | 92 | 0.63 | 0.38 – 0.88 | <0.001 |
| | | Total (n=769) | 91 | 0.66 | 0.59 – 0.73 | <0.001 |

**Table S2.** Proportions of participants with recordings with quality, recordings with adventitious sounds, and stethoscope identified adventitious sounds by location, age group and diagnostic group.

| | | | **With quality or adventitous sounds** | **No quality or no adventitious sounds** | **Proportion (%)** |
|---|---|---|---|---|---|
| **Recordings with quality** | Age group | Children (n=92) | 84 | 8 | 95 |
| | | Adults (n=42) | 39 | 3 | 93 |
| | Diagnostic group | Cystic Fibrosis (n=42) | 39 | 3 | 93 |
| | | Other Respiratory Diseases (n=39) | 37 | 2 | 95 |
| | | Asthma (n=37) | 32 | 5 | 87 |
| | | No Respiratory Diseases (n=16) | 15 | 1 | 94 |
| | | Total (n=134) | 123 | 11 | 92 |
| **Recordings with adventitious sounds** | Age group | Children (n=84) | 29 | 55 | 35 |
| | | Adults (n=39) | 14 | 25 | 36 |
| | Diagnostic group | Cystic Fibrosis (n=39) | 10 | 29 | 26 |
| | | Other Respiratory Diseases (n=37) | 20 | 17 | 54 |
| | | Asthma (n=32) | 9 | 23 | 28 |
| | | No Respiratory Diseases (n=15) | 4 | 11 | 27 |
| | | Total (n=123) | 43 | 80 | 35 |
| **Stethoscope identified adventions sounds** | Age group | Children (n=84) | 12 | 72 | 14 |
| | | Adults (n=39) | 20 | 19 | 51 |
| | Diagnostic group | Cystic Fibrosis (n=39) | 14 | 25 | 36 |
| | | Other Respiratory Diseases (n=37) | 18 | 19 | 49 |
| | | Asthma (n=32) | 0 | 32 | 0 |
| | | No Respiratory Diseases (n=15) | 0 | 15 | 0 |
| | | Total (n=123) | 32 | 91 | 26 |

**Table S3.** Comparison of the proportions of participants with recordings with quality, recordings with adventitious sounds, and stethoscope identified adventitious sounds between age group or diagnostic group.

| | | Comparison | | Chi-Square |
|---|---|---|---|---|
| **Recordings with quality** | Age group | Children | Adults | 0.761 |
| | Diagnostic group | Diagnostic group overall comparison | | 0.569 |
| **Recordings with adventitious sounds** | Age group | Children | Adults | 0.882 |
| | Diagnostic group | Diagnostic group overall comparison | | 0.036* |
| | | Cystic Fibrosis | Other Respiratory Diseases | 0.011 |
| | | Cystic Fibrosis | Asthma | 0.814 |
| | | Cystic Fibrosis | No Respiratory Diseases | 0.939 |
| | | Other Respiratory Diseases | Asthma | 0.030 |
| | | Other Respiratory Diseases | No Respiratory Diseases | 0.073 |
| | | Asthma | No Respiratory Diseases | 0.917 |
| **Stethoscope identified adventions sounds** | Age group | Children | Adults | <0.001* |
| | Diagnostic group | Diagnostic group overall comparison | | <0.001* |
| | | Cystic Fibrosis | Other Respiratory Diseases | 0.260 |
| | | Cystic Fibrosis | Asthma | <0.001* |
| | | Cystic Fibrosis | No Respiratory Diseases | 0.007* |
| | | Other Respiratory Diseases | Asthma | <0.001* |
| | | Other Respiratory Diseases | No Respiratory Diseases | <0.001* |
| | | Asthma | No Respiratory Diseases | † |

* A statistically significant difference was found.

† Chi-Square could not be calculated because the stethoscope's findings were constant in both groups (no adventitious sounds were found).

**Table S4.** Comparison of the proportions of recordings with quality with adventitious sounds between auscultation location, age group or diagnostic group.

| | | Comparison | | Chi-Square |
|---|---|---|---|---|
| Quality | Location | Auscultation location overall comparison | | <0.001* |
| | | Trachea | Right Anterior Chest | 0.007* |
| | | Trachea | Right Posterior Base | <0.001* |
| | | Trachea | Left Posterior Base | 0.003* |
| | | Right Anterior Chest | Right Posterior Base | 0.069 |
| | | Right Anterior Chest | Left Posterior Base | 0.782 |
| | | Right Posterior Base | Left Posterior Base | 0.125 |
| | Age group | Children | Adults | 0.020* |
| | Diagnostic group | Diagnostic group overall comparison | | 0.052 |
| Presence of adventitious sounds | Location | Auscultation location overall comparison | | 0.420 |
| | Age group | Children | Adults | 0.211 |
| | Diagnostic group | Diagnostic group overall comparison | | <0.001* |
| | | Cystic Fibrosis | Other Respiratory Diseases | <0.001* |
| | | Cystic Fibrosis | Asthma | 0.208 |
| | | Cystic Fibrosis | No Respiratory Diseases | 0.312 |
| | | Other Respiratory Diseases | Asthma | <0.001* |
| | | Other Respiratory Diseases | No Respiratory Diseases | <0.001* |
| | | Asthma | No Respiratory Diseases | 0.989 |

**Table S5.** Comparison between the findings of conventional auscultation and smartphone auscultation regarding the participants and the recordings.

| | | | Agreement % | Cohens' k | 95% CI | p |
|---|---|---|---|---|---|---|
| Participants | Age group | Children (n=84) | 70 | 0.24 | 0.04 – 0.44 | 0.011 |
| | | Adults (n=39) | 54 | 0.084 | -0.21 – 0.38 | 0.584 |
| | Diagnostic group | Cystic Fibrosis (n=39) | 59 | 0.049 | -0.26 – 0.36 | 0.754 |
| | | Other Respiratory Diseases (n=37) | 62 | 0.25 | -0.06 – 0.56 | 0.134 |
| | | Asthma (n=32) | 72 | 0† | | |
| | | No Respiratory Diseases (n=15) | 73 | 0† | | |
| | | Total (n=123) | 65 | 0.18 | 0 – 0.36 | 0.038 |
| Recordings | Location | Trachea (n=223) | 85 | 0.052 | -0.08 – 0.19 | <0.001 |
| | | Right Anterior Chest (n=189) | 86 | 0.46 | 0.29 – 0.63 | <0.001 |
| | | Right Posterior Base (n=173) | 82 | 0.36 | 0.18 – 0.54 | <0.001 |
| | | Left Posterior Base (n=184) | 88 | 0.46 | 0.28 – 0.64 | <0.001 |
| | Age group | Children (n=531) | 87 | 0.35 | 0.23 – 0.47 | <0.001 |
| | | Adults (n=238) | 81 | 0.34 | 0.19 – 0.49 | <0.001 |
| | Diagnostic group | Cystic Fibrosis (n=273) | 83 | 0.094 | -0.06 – 0.24 | 0.091 |
| | | Other Respiratory Diseases (n=231) | 81 | 0.53 | 0.40 – 0.66 | <0.001 |
| | | Asthma (n=183) | 90 | 0† | | |
| | | No Respiratory Diseases (n=92) | 90 | 0† | | |
| | | Total (n=769) | 85 | 0.35 | 0.26 – 0.44 | <0.001 |
